# Understanding for whom, under what conditions and how smoking cessation services for pregnant women in the United Kingdom work—a rapid realist review

**DOI:** 10.1186/s12889-023-17378-w

**Published:** 2023-12-12

**Authors:** Claire Tatton, Jenny Lloyd

**Affiliations:** Faculty of Health and Life Sciences, Department of Health and Community Sciences, University of Exeter, Exeter, UK

**Keywords:** Smoking cessation, Pregnancy, United Kingdom, Realist review

## Abstract

**Background:**

Maternal smoking in pregnancy is associated with several adverse maternal and infant health outcomes including increased risk of miscarriage, stillbirth, low birth weight, preterm birth, and asthma. Progress to reduce rates of smoking at time of delivery in England have been slow and over the last decade, less than half of pregnant women who accessed services went onto report having quit. This realist review was undertaken to improve the understanding of how smoking cessation services in pregnancy work and to understand the heterogeneity of outcomes observed.

**Methods:**

The initial programme theory was developed using the National Centre for Smoking Cession and Training Standard Treatment Programme for Pregnant Women and the National Institute for Health and Care Excellence guidance on treating tobacco dependency. A search strategy and inclusion criteria were developed. Four databases were searched to identify published papers and four websites were hand searched to identify any unpublished literature that could contribute to theory building. Realist logic was applied to the analysis of papers to identify the contexts in which the intended behaviour change mechanism(s) were triggered, or not, and towards what outcomes to develop context mechanism outcome configurations.

**Results:**

The review included 33 papers. The analysis produced 19 context mechanism outcome configurations structured under five closely interconnected domains (i) articulating harm, (ii) promoting support, (iii) managing cravings, (iv) maintaining commitment and (v) building self-efficacy. This review identifies two key processes involved in how services achieve their effects: how material resources are implemented and relationships. Of the two key processes identified, more existing literature was available evidencing how material resources are implemented. However, the review provides some evidence that non-judgemental and supportive relationships with healthcare workers where regular contact is provided can play an important role in interrupting the social cues and social practice of smoking, even where those around women continue to smoke.

**Conclusions:**

This review clarifies the range of interconnected and bi-directional relationships between services and the personal and social factors in women’s lives. It underscores the importance of aligning efforts across the models five domains to strengthen services’ ability to achieve smoking cessation.

**Supplementary Information:**

The online version contains supplementary material available at 10.1186/s12889-023-17378-w.

## Background

Smoking remains the largest contributor to illness and premature mortality in the UK [1]. Maternal smoking during pregnancy is a continued public health concern due to the adverse outcomes on maternal and infant health including increased risk of miscarriage, ectopic pregnancy, sudden infant death syndrome, stillbirth, low birth weight, preterm birth, and asthma [2, 3]. Maternal smoking during pregnancy also presents potential long-term adverse health outcomes to infants including increased risk of overweight and obesity and intellectual impairment which may not be observed until later in life [2, 3]. In high-income countries, smoking in pregnancy is socially patterned and strongly associated with socioeconomic disadvantage [3]. Those who smoke in pregnancy are more likely to have started smoking early in life, to experience financial hardship and to have partners and/or social networks who are more likely to smoke [3].

Smoking at time of delivery rates (SATOD) in England have been slowly declining, falling from 15.8% in 2006/07 to 8.8% in 2022/23 [4]. However, the rate of decline has missed the UK Government’s ambition to reach a rate of 6% SATOD, or less, by 2022 [5]. Revised predictions now suggest that this ambition will not be reached until 2032 [6]. Whilst many women may attempt to stop smoking in pregnancy, they can experience physical and psychological barriers from doing so [3]. Data from smoking cessation services in England from 2010/11 to 2021/22 show that every year, less than half of women who engage with services and set a quit date went onto record a successful quit [7].

Research conducted to date has focussed on understanding the effectiveness of single interventions, different service configurations and staff training used throughout the UK and on understanding women’s experiences using qualitative approaches. A health technology assessment (HTA) published in 2017 [3] developed and synthesised literature across multiple interventions and perspectives to understand the many barriers and facilitators women experience in their attempts to stop smoking during pregnancy. The review found that women’s smoking cessation perceptions and experiences were fluid and context dependent, acting as either barriers or facilitators to quitting. Important factors were women’s belief about the harm of smoking in pregnancy, their changing relationship with their baby throughout pregnancy, the nature of the relationships with their partner, friends and family members, their own sense of psychological wellbeing and their belief in their ability to quit. These factors impacted on each other in non-linear patterns and changed overtime throughout pregnancy. Within a number of studies included in the review, the factors involved in achieving smoking cessation were explored through behaviour change frameworks. The COM-B system was most commonly used. The COM-B system proposes there are three sources of behaviour (capability, opportunity, and motivation) which need to be present for a particular behaviour to occur. Each source comprises two elements—capability (psychological and physical), opportunity (physical and social) and motivation (reflective and automatic) [8]. The review concluded that, to be effective, interventions should take account of the interplay between factors working across different aspects of women’s lives [3].

### Objectives and focus of the review

The present review will build on these findings by framing smoking as a social practice to better understand how an individual’s interactions with their social networks, and smoking cessation services might form, reinforce, and change smoking behaviour [9].

The aim of this review is to improve the understanding of how smoking cessation services in the UK to reduce smoking in pregnancy work, for whom, and under what circumstances. The objectives are to (i) use realist principles to synthesise a range of existing literature across multiple smoking cessation interventions to develop an understanding of how services work, (ii) understand and describe in which contexts behaviour change mechanisms are or are not triggered by services, and the resulting outcomes, and (iii) provide recommendations for policy and practice.

### Rationale for realist review approach

Realist reviews (or realist synthesis) are explanatory and strive to unpack how, why, for whom and in what contexts policies and programmes work or do not work. Realist reviews, therefore, resist the notion of generalisability and give more value to explanatory theories about how policies and programmes are shaped by context. This is done by theorising on the underlying mechanisms that may explain why and how change occurs. These programme theories are developed, refined and tested through data provided by the review's included sources [10, 11]. By utilising a diverse range of literature, the explanatory model generated is likely to have greater generalisability than a model generated from a single setting or approach. Therefore, a realist review is beneficial for the analysis of complex social programmes, such as smoking cessation services, producing findings beneficial to researchers, policymakers, and practitioners.

## Methods

### Review process

This review is based on Pawson’s five iterative stages [12]. The reporting of this review is consistent with the RAMESES publication standards and reporting for realist reviews [12, 13].

#### Scoping the literature

The initial programme theory (a set of theoretical explanations or assumptions about how a programme is expected to work) [12] was built using the National Centre for Smoking Cession and Training (NCSCT) Standard Treatment Programme for Pregnant Women [14] and the National Institute for Health and Care Excellence (NICE) guidance on treating tobacco dependency [15]. Together these comprise recommended evidence-based interventions and approaches (referred to throughout as delivery components) for use in the UK. Consistent with approaches taken in existing literature in the field, we used the COM-B model of behaviour change as the framework for the initial programme theory. Intervention delivery components were mapped to each behaviour source (capability, opportunity, and motivation) within this model [8] to understand the theorised mechanisms and outcomes. Potential sources of resistance were drawn from the NCSCT Programme and NICE guidance [14, 15]. The initial programme theory is set out in Table 1.

**Table 1:** Initial programme theory

Delivery component	Theorised mechanism	Anticipated outcome	Potential resistance
Inform and reinforce that smoking in pregnancy is harmful	Psychological capability	Health risks are understood and believed	Alternative messages form belief that smoking isn’t harmful, health risks are exaggerated or cutting down is sufficient
	Reflective motivation	Belief of health risk results in planning and committing to stop smoking	Perceived benefits of smoking in pregnancy – smaller baby to deliver Concern about negative health impacts resulting from stopping smoking—weight gain or increased stress
Carbon monoxide (CO) monitoring	Psychological capability	Improved understanding of harm	Low reading can give false belief of low risk
“Opt out” referral system to stop smoking service	Physical opportunity	All pregnant women are offered the opportunity and support to stop smoking	Stigma of smoking in pregnancy may lead to non-disclosure or underreporting of smoking Unsuitable or inaccessible services
	Reflective motivation	Knowledge of harm and available support leads to planning to quit	Low self-belief of ability to quit Concerns of coping without smoking
Educate about the safety of nicotine replacement therapy (NRT) and vapes (quit aids)	Psychological capability	Use in pregnancy is viewed as acceptable and aids women to stop smoking	Alternative messages that create or reinforce belief of harm Stigma and judgement from others of using them
Provide access to quit aids	Physical capability	Adherence to smoking cessation is improved through receiving a clean form of nicotine to manage cravings	Increased nicotine metabolism in pregnancy makes cravings stronger and more difficult to manage Unpleasant taste and/or skin irritation
	Physical opportunity	Alternative products to manage cravings reduce temptation to smoke	Cost or lack of easy access
	Automatic motivation	Desire for nicotine is met by alternative, safer products	Stigma and judgement from others for using a vape
Provide positive, non-judgemental behavioural support	Physical opportunity	Regular contact motivates and improves self-efficacy to quit	Service is difficult to access
	Reflective motivation	Individualised coping strategies mean readiness to respond to cravings or triggers to smoke	Concern of being judged for not being able to quit Low self-efficacy
Offer support to partner or supporter to quit smoking	Social opportunity	Social support and encouragement to quit attempt	Tension in relationship over smoking
	Automatic motivation	Reduces triggers to smoke	Partner / significant other chooses not to quit or is unable to
Offer financial incentives and use regular CO monitoring to validate	Reflective motivation	Seeing progress builds motivation and self-efficacy	Concern of being judged for not being able to quit

#### Searching process

An initial search strategy, search terms and inclusion criteria were developed from background reading [3, 14, 15]. Search terms were tested in Embase (accessed via Ovid). The strategy was developed iteratively using the titles and abstracts of papers retrieved to identify additional or alternative terms and synonyms. Several versions of the search strategy were tested to minimise retrieval of irrelevant studies and to test that initially identified relevant studies continued to be retrieved. The final search was conducted in April 2023. The inclusion criteria are set out in Table 2 and final search strategy is available in Additional file 1. The databases CAB Abstracts, Embase, Global Health, OVID MEDLINE(R) were searched via Ovid. Search results were de-duplicated in Ovid using the automated de-duplication function and results were exported to Microsoft Excel for manual checking.

**Table 2:** Inclusion criteria

	**Inclusion criteria**	**Exclusion criteria**
**Population**	Pregnant women who smoke in the UK	• Women who smoke pre-conception or post-natal • Pregnant women exposed to second hand smoke
**Intervention**	Interventions in the UK delivered directly to pregnant women where the stated aim is to “stop smoking”	• General smoking interventions where the delivery and outcomes specific to pregnant women cannot be identified • Integrated health behaviour interventions where delivery and outcomes specific to smoking cessation in pregnancy cannot be identified • Smoking cessation interventions delivered alongside interventions and support for multiple complex needs (alcohol dependency, mental health, domestic abuse)
**Outcomes**	• Smoking cessation service enrolment • Smoking quit attempts, quit achieved or reduction • Reported barriers and facilitators to smoking cessation or reduction	• Infant outcomes • Family outcomes • Studies where smoking status is measured as a confounder or predictor for other health outcomes
**Study designs**	• Empirical studies including qualitative, quantitative, and mixed methods • Service evaluations or case studies	• Economic analyses • Study protocols
**Other data sources**	• Reported barriers and facilitators to smoking cessation • Perceptions or experiences of pregnant women and healthcare professionals about smoking in pregnancy or smoking cessation interventions • Representation of smoking cessation interventions in the media	Public perceptions or surveys of smoking cessation interventions
**Article language**	English	Other languages
**Dates**	Published between 2010—2023	Published before 2010

A historical date limit of 2010 was applied to the search. In 2010, NICE published the first guidelines about how to stop smoking in pregnancy including recommendations of evidence-based interventions and approaches [16, 17]. Therefore, studies pre-dating 2010 were considered to potentially be an inaccurate representation of current provision.

Handsearching was also conducted to identify any unpublished literature that could contribute to theory building. Websites searched were the Local Government Association, Gov.uk, Action on Smoking and Health (ASH) and the Kings Fund.

#### Selection and appraisal of documents

Detailed inclusion and exclusion criteria were developed by CT and JL. CT carried out the initial abstract and title screening to exclude studies outside of the scope of the review. Full text screening was undertaken in two stages. The first screening assessed papers against the inclusion criteria and the second screening appraised papers based on their relevance and rigour. In the first stage, JL rescreened 5 included studies to check appropriate application of the inclusion criteria, after which any uncertainties/discrepancies were discussed before a final agreement on included studies was made. In the second stage, JL rated relevance and rigour for 10% of the sample (blinded). Any disagreements were discussed, before a final rating was agreed. The rigour and relevance of all other studies was discussed in meetings before a final rating was agreed.

Relevance was assessed through criteria developed for this review by judging the studies ability to contribute to theory building through (i) similarity of intervention to the initial programme theory or of other search results (ii) understanding of the different level(s) (individual, interpersonal, organisational, social) the service operated at or was experienced at, and (iii) understanding of the mechanisms that the service intended to trigger or that were observed. Papers were coded as highly relevant, somewhat relevant, of limited relevance or not relevant depending on the strength of evidence presented. Papers assessed as not relevant were excluded.

In accordance with the realist approach, rigour was assessed according to the design of each study type, as opposed to their position in the hierarchy of evidence [10]. Included papers were coded as high, medium, or low quality based on the assessment of (i) the appropriateness of the study design to the research question/aims used, (ii) methodological rigour of the selected study design, and (iii) evidence of critical analysis of the findings. Assessment of papers against the criteria developed for this review was guided by available tools and checklists. For randomised controlled trials, qualitative studies and the systematic review included in this review, the prompts in the Critical Appraisal Skills Programme (CASP) checklists for randomised controlled trials, qualitative studies, and systematic review were used, respectively [18]. For mixed methods studies, assessment was guided by Pluye et al.’s scoring system for appraising quality of mixed methods research [19]. For other study types including surveys, a literature review, a service evaluation and the non-peer reviewed literature, assessment of rigour was guided by Pawson’s approach [20]. These other study designs were assessed according to the following questions (i) are the details of the methods used clearly reported, including reflection of the potential limitations of the method selected? (ii) are the study sample size, data collection and data analysis techniques appropriate for the objective of the study? and (iii) are the conclusions drawn reasonable and justified in the context of the limitations of the method used? The study level responses using the various checklists used are reported in Additional file 2.

CT categorised the papers as primary or secondary papers depending on their appraisal ratings with critical guidance from JL to agree the categorisation. In keeping with the realist approach, studies assessed as having low methodological rigour were not necessarily excluded from this review [20]. However, these studies were categorised as secondary papers and were therefore used to test and refine the findings of primary studies which demonstrated high methodological rigour. The full lists of included papers are reported in Tables 3 and 4, and study level appraisal ratings are included in Additional file 2.

**Table 3:** Primary studies included in this review

Title, author, date and reference	Study design	Delivery components studied	Participants studied	Number of participants
McKell. J et al. (2022) [21] *Usual care in a multicentre randomised controlled trial of financial incentives for smoking cessation in pregnancy: qualitative findings from a mixed methods process evaluation* http://dx.doi.org/10.1136/bmjopen-2022-066494	Qualitative	CO monitoring, opt-out referrals, financial incentives, behavioural support, and provision of quit aids Behavioural support differed across sites. Support was delivered by telephone or face to face appointments, in the hospital, community or by home visits. In most instances, at least one face-to-face counselling session with follow-up support was provided to 12 weeks after a quit date was set	Pregnant smokers, midwives and stop smoking advisors	51
McCormack F.C et al. (2022) [22] *Exploring pregnant women’s experiences of stopping smoking with an incentive scheme with 'enhanced' support: a qualitative study.* https://doi.org/10.1177/17579139221106842	Qualitative	Financial incentives, provision of quit aids, CO monitoring and behavioural support Behavioural support was described as contact from stop smoking advisors, at least 4-weekly, on a one-to-one basis throughout pregnancy and for 12 weeks after. Type(s) of contact and venue(s) used were not reported	Pregnant women and stop smoking advisors	15
Stacey T. et al. (2022) [23] *‘I don’t need you to criticise me, I need you to support me’. A qualitative study of women’s experiences of and attitudes to smoking cessation during pregnancy* https://doi.org/10.1016/j.wombi.2022.01.010	Qualitative	Education of harm, education of safety and effectiveness of quit aids and provision of quit aids	Pregnant smokers	19
Griffiths S.E et al. (2022) [24] *Accessing specialist support to stop smoking in pregnancy: A qualitative study exploring engagement with UK-based stop smoking services.* https://doi.org/10.1111/bjhp.12574	Qualitative	Education of harm, opt-out referrals and behavioural support Behavioural support was described as delivered in the community, predominantly on a one-to-one basis at home visits. Regularity of contact was not reported	Pregnant smokers, pregnant women who had recently quit smoking, midwives and stop smoking advisors	28
McDaid L. et al. (2021) [25] *Understanding pregnant women's adherence-related beliefs about Nicotine Replacement Therapy for smoking cessation: A qualitative study.* https://doi.org/10.1111/bjhp.12463	Qualitative	Education of safety and effectiveness of quit aids and provision of quit aids	Pregnant smokers	18
Froggatt S. et al. (2021) [26] *Risk perception of cigarette and e-cigarette use during pregnancy: A qualitative postpartum perspective* https://doi.org/10.1016/j.midw.2020.102917	Qualitative	Education of safety and effectiveness of quit aids and education of harm	Pregnant smokers	14
Hunter A. et al. (2021) [27] *Healthcare Professionals’ Beliefs, Attitudes, Knowledge, and Behaviour around Vaping in Pregnancy and Postpartum: A Qualitative Study.* https://doi.org/10.1093/ntr/ntaa126	Qualitative	Education of safety and effectiveness of quit aids and provision of quit aids	Midwives, health visitors, GPs and stop smoking advisors	60
Campbell K. et al. (2020) [28] *Factors influencing the uptake and use of nicotine replacement therapy and e-cigarettes in pregnant women who smoke: a qualitative evidence synthesis.* https://doi.org/10.1002/14651858.CD013629	Systematic review	Education of safety and effectiveness of quit aids and education of harm	Pregnant smokers and women post-partum who had smoked throughout pregnancy	497
Grant A. et al. (2020) [29] *Smoking during pregnancy, stigma and secrets: Visual methods exploration in the UK.* https://doi.org/10.1016/j.wombi.2018.11.012	Qualitative	Education of harm and education of safety and effectiveness	Pregnant smokers	10
Thomson R. et al.(2019) [30] *Knowledge and education as barriers and facilitators to nicotine replacement therapy use for smoking cessation in pregnancy: A qualitative study with health care professionals.* https://doi.org/10.3390/ijerph16101814	Qualitative	Education of safety and effectiveness and provision of quit aids	Stop smoking advisors, antenatal care staff	26
Naughton F. et al. (2018) [31] *Barriers and facilitators to smoking cessation in pregnancy and in the post-partum period: The health care professionals’ perspective.* https://doi.org/10.1111/bjhp.12314	Qualitative	Education of harm, CO monitoring, opt-out referrals, education of safety and effectiveness and provision of quit aids	Midwives, obstetricians, health visitors, GPs, pharmacists, commissioners, stop smoking service advisors and managers	48
Campbell K.A. et al. (2016) [32] *Antenatal clinic and stop smoking services staff views on "opt-out" referrals for smoking cessation in pregnancy: A framework analysis.* https://doi.org/10.3390/ijerph13101004	Qualitative	CO monitoring and opt-out referrals	Antenatal staff and stop smoking advisors	11
Sloan M. et al. (2016) [33] *Pregnant Women’s Experiences and Views on an “Opt-Out” Referral Pathway to Specialist Smoking Cessation Support: A Qualitative Evaluation* https://doi.org/10.1093/ntr/ntv273	Qualitative	Education of harm, CO monitoring and opt-out referrals	Pregnant smokers	18
Bauld L. et al. (2017) [3] *Barriers to and facilitators of smoking cessation in pregnancy and following childbirth: Literature review and qualitative study* https://doi.org/10.3310/hta21360	Qualitative	Education of harm, CO monitoring, opt-out referrals, education of safety and effectiveness, provision of quit aids and behavioural support Behavioural support was described as more frequently provided on a one-to-one basis than group provision and in a range of venues. In most cases, support was offered throughout pregnancy, and in some cases, after birth. However, no specific reporting on regularity of contact was provided	Pregnant smokers, postpartum women who had smoked through pregnancy, significant others of pregnant smokers and Healthcare professionals	121
Bluegrass. (2022) [34] *Action on Smoking and Health: Qualitative insights.* https://ash.org.uk/uploads/Qualitative_Insights_Primary_Research_Report_2022-12-02-140553_trte.pdf?v=1669989950	Qualitative	Education of harm, CO monitoring, opt-out referrals and education of safety and effectiveness	Pregnant smokers	6

**Table 4:** Secondary studies included in this review

Title, author, date and reference	Study design	Delivery components studied	Participants studied	Number of participants
Grant A. et al. (2019) [35] *Understanding health behaviour in pregnancy and infant feeding intentions in low-income women from the UK through qualitative visual methods and application to the COM-B model.* https://doi.org/10.1186/s12884-018-2156-8	Qualitative	Education of harm	Pregnant women living in low-income areas	10
Cooper S. et al. (2019) [36] *Attitudes to E-cigarettes and cessation support for pregnant women from English stop smoking services: A mixed methods study.* https://doi.org/10.3390/ijerph16010110	Mixed methods	Education of safety and effectiveness of quit aids, provision of quit aids and behavioural support Behavioural support was most often provided on a one-to-one basis in clinic settings. One-to-one support in homes was also provided in most services and was associated with higher levels of take-up by women. Telephone, email, and text support was offered less frequently and had low take-up rates. Regularity of contact was not reported	Stop smoking service managers	72
Bowker K. et al. (2018) [37] *Views on and experiences of electronic cigarettes: A qualitative study of women who are pregnant or have recently given birth.* https://doi.org/10.1186/s12884-018-1856-4	Qualitative	Education of safety and effectiveness of quit aids and provision of quit aids	Pregnant and post-partum women who were current smokers or had recently quit	30
Crossland N. et al. (2015) [38] *Incentives for breastfeeding and for smoking cessation in pregnancy: An exploration of types and meanings.* https://doi.org/10.1016/j.socscimed.2014.12.019	Qualitative	Financial incentives	Pregnant women, significant others, service providers, and decision makers	165
Thomson G. et al. (2014) [39] *Unintended consequences of incentive provision for behaviour change and maintenance around childbirth.* https://doi.org/10.1371/journal.pone.0111322	Mixed methods	Financial incentives	Pregnant and post-partum women, significant others, service providers, policymakers, and healthcare professionals	674
Mantzari E. et al. (2012) [40] *The effectiveness of financial incentives for smoking cessation during pregnancy: Is it from being paid or from the extra aid?* https://doi.org/10.1186/1471-2393-12-24	Qualitative	Financial incentives, CO monitoring and provision of quit aids	Pregnant smokers	36
Moyse M. et al. (2021) [41] Newspaper *media representation of electronic cigarette use during pregnancy.* https://doi.org/10.1093/pubmed/fdaa048	Literature review	Education of safety and effectiveness of quit aids	N/A	N/A
Forman J. et al. (2017) [42] *National survey of smoking and smoking cessation education within UK Midwifery School curricula.* https://doi.org/10.1093/ntr/ntw230	Survey	Education of harm and education of safety and effectiveness of quit aids	Midwifery schools	29
O'Connell M & Duaso M(2014) [43] *Barriers and facilitators of midwives' use of the carbon monoxide breath test for smoking cessation in practice: a qualitative study.* https://www.researchgate.net/publication/269699226_Barriers_and_facilitators_of_midwives'_use_of_the_carbon_monoxide_breath_test_for_smoking_cessation_in_practice_a_qualitative_study	Qualitative	CO monitoring and opt-out referrals	Midwives	10
Bowker K et al. (2021) [44] *Pregnant women's use of e-cigarettes in the UK: a cross-sectional survey*. https://doi.org/10.1111/1471-0528.16553	Survey	Education of safety and effectiveness of quit aids and provision of quit aids	Pregnant smokers	3360
Naughton F. et al. (2020) [45] *Interest in and Use of Smoking Cessation Support across Pregnancy and Postpartum.* https://doi.org/10.1093/ntr/ntz151	Survey	Education of harm and behavioural support Behavioural support accessed was described as delivered through group sessions, one-to-one appointments, or telephone helpline. Regularity of contact was not reported	Pregnant smokers and recent ex-smokers	850
Vaz L.R. et al. (2017) [46] Factors associated with the effectiveness and reach of NHS stop smoking services for pregnant women in England. https://doi.org/10.1186/s12913-017-2502-y	Survey	Behavioural support and financial incentives Behavioural support delivered in clinic settings compared to home visits was reported as associated with greater reach and effectiveness. Regularity of contact was not reported	NHS Stop smoking service data	121
Campbell K.A. et al. (2016) [32] *‘Opt-out’ referrals after identifying pregnant smokers using exhaled air carbon monoxide: impact on engagement with smoking cessation support.* http://dx.doi.org/10.1136/tobaccocontrol-2015-052662	Before – after evaluation	CO monitoring and opt-out referrals	NHS Stop smoking service data	2300
Beenstock J. et al. (2012) [47] *What helps and hinders midwives in engaging with pregnant women about stopping smoking? A cross-sectional survey of perceived implementation difficulties among midwives in the North East of England.* https://doi.org/10.1186/1748-5908-7-36	Survey	Education of harm, CO monitoring and opt-out referrals	Midwives	364
Tappin D. et al. (2022) [48] *Effect of financial voucher incentives provided with UK stop smoking services on the cessation of smoking in pregnant women (CPIT III): Pragmatic, multicentre, single blinded, phase 3, randomised controlled trial.* https://doi.org/10.1136/bmj-2022-071522	Randomised control trial	Financial incentives	Trial data of pregnant smoker’s cessation outcomes	944
Tappin D. et al. (2015) [49] *Financial incentives for smoking cessation in pregnancy: Randomised controlled trial.* https://doi.org/10.1136/bmj.h134	Randomised control trial	Financial incentives	Trial data of pregnant smoker’s cessation outcomes	612
Thomson R. et al. (2022) [50] *Smoking Cessation Support for Pregnant Women Provided by English Stop Smoking Services and National Health Service Trusts: A Survey.* https://doi.org/10.3390/ijerph19031634	Survey	CO monitoring, behavioural support, and provision of quit aids Behavioural support was delivered similarly across Local Authority and NHS settings. The most common type was one-to-one support delivered remotely, by either telephone or video call. Regularity of contact was not reported	Tobacco Control Leads, commissioners, smoking cessation specialist midwives and team leaders	194
Local Government Association (2018) [51] *Fit for and during pregnancy: a key role for local government* https://www.local.gov.uk/sites/default/files/documents/15.52%20Fit%20for%20and%20during%20pregnancy_03.pdf	Case studies	Education of harm, CO monitoring, opt-out referrals, financial incentives, and provision of quit aids	Pregnant smokers, midwives and stop smoking advisors	Not reported

#### Data extraction

Explanations of causation in realist reviews are expressed as context mechanism outcome configurations (CMOCs) [10, 52]. Realist logic was applied to the analytic process which sought to identify contextual factors relating to whether an intervention triggered the behaviour change mechanism(s) intended, and the outcomes. Data was initially organised in a diagram (see Fig. 2) to depict the relationships between interlinked delivery components within services, and factors across different levels of women's lives (individual, interpersonal, organisational, and societal) that impact how services are delivered or experienced. This supported greater understanding of the data and highlighted key relationships. Relevant data for theory building were extracted from included studies and recorded in a data extraction template developed in Microsoft Excel for this review to generate an initial list of CMOCs. The data extraction template was developed and tested by CT and JL through the process of developing the initial programme theory. Inductive reasoning was used to classify CMOCs according to the primary mechanism (capability, opportunity, or motivation) it intended to trigger.

#### Analysis and synthesis process

Data synthesis was conducted by CT with critical guidance from JL. CMOCs were tested and developed iteratively through constant comparison and through discussion between CT and JL. Primary studies were first compared to identify common confirmatory accounts of how interventions worked to bring about their effects to develop an initial set of CMOCs. Contrasting accounts from primary studies were used to refine the CMOCs. The same process was undertaken with the secondary studies. CMOCs derived from secondary studies were used to test and further refine the CMOCs derived from the primary studies. These were compared with the initial programme theory to produce the final set of best evidenced CMOCs and to draw conclusions.

## Results

**Fig. 1:**
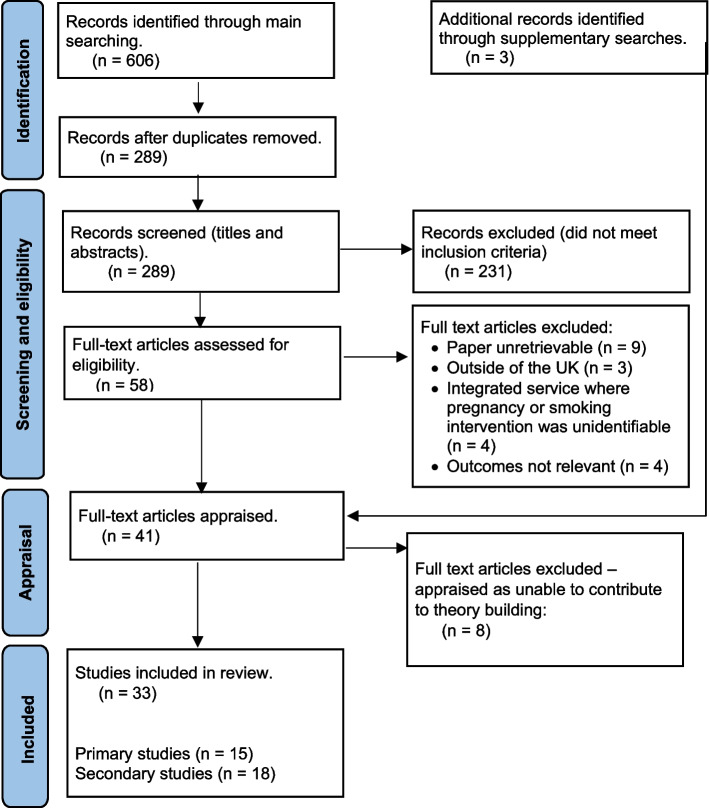
Flow diagram of the search and results

The initial search identified 609 records, 38 were retrieved from searching bibliographic databases which met the inclusion criteria, and an additional 3 papers were identified through handsearching. 33 studies were included in the final review [3, 21–51, 53] (see Fig. 1 for the PRISMA flowchart).

### Document characteristics

The 33 papers comprise 20 qualitative studies [3, 21–27, 29–35, 37, 38, 40, 43], one systematic review [28] five surveys [42, 44–47], two mixed-methods studies [36, 39], two randomised controlled trials [48, 49], one service evaluation [53], one case study report [51] and one literature review [41]. Twelve studies were published between 2010 – 2017 and 21 studies were published from 2018 onwards. The full details and characteristics of papers included in this review are reported in Tables 3 and 4.

Interventions studied in included papers were carbon monoxide (CO) monitoring and opt-out referrals to local stop smoking services, financial incentives, provision of, and use of quit aids (nicotine replacement therapy and vapes/e-cigarettes). Included papers also covered the approaches taken by healthcare professionals to engage women in smoking cessation services, including their training, and the perceptions of women about their experiences with healthcare professionals and services.

Overall, the quality and relevance of the included studies was high. The large number of qualitative studies included in this review contributed a depth of understanding relevant to theory building regarding the factors at different levels of women’s lives that impact how smoking cessation services are experienced. Whilst these studies often had a low number of participants, the rigour of the approaches used, and the number of studies included provide assurance of the relevance of the findings over different intervention settings. Full details of the appraisal judgements of included studies are provided in Additional file 2.

**Fig. 2:**
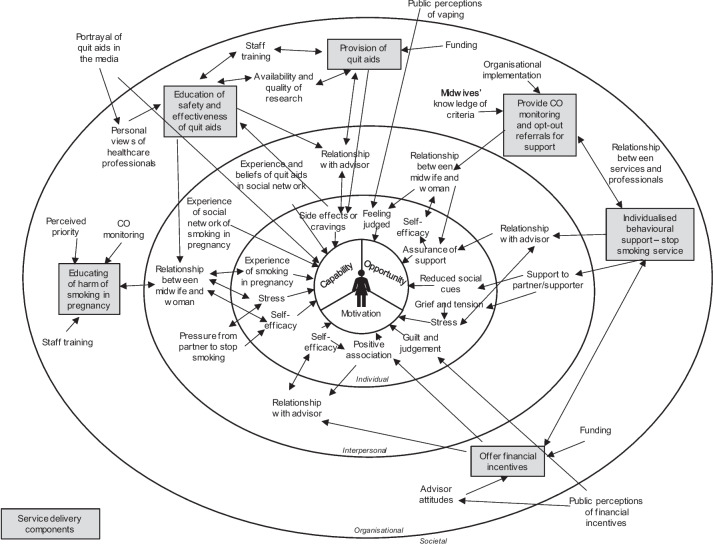
Key relationships

Figure 2 depicts the key relationships between delivery components within services and the factors across different levels of women's lives (individual, interpersonal, organisational, and societal) that impact how services are delivered and experienced.

### Main findings

The analysis developed 19 CMOCs, structured across five domains (i) articulating harm, (ii) promoting support, (iii) managing cravings, (iv) maintaining commitment and (v) building self-efficacy. Domains relate to the COM-B sources of behaviour—capability, motivation, and opportunity [8]. Table 5 provides a summary of the 19 CMOCs which make up the programme theory. The domains are closely interconnected meaning that the ability of services to achieve their intended outcomes is strengthened by aligning efforts across all five domains.

**Table 5:** Summary of CMOCs (programme theory)

CMOC	Description
***Articulating harm***	
CMOC1	In organisations where smoking cessation training is unavailable, not mandated, or insufficient (C) midwives can feel unconfident, unmotivated, or unable to discuss smoking (M) resulting in mixed messages about harm and the importance of smoking cessation (O)
CMOC2	Where midwives are concerned about being perceived as judgemental or are aware of women’s views on smoking (C) midwives can feel unconfident, unable, or unwilling to discuss smoking (M) leading to missed opportunities to influence women’s views or diluted messages that avoid causing potential distress or damaging the relationship (O)
CMOC3	Where women have personal experience of smoking in pregnancy or have women in their social network who have (C), they assess the health of the children born to be tangible, realistic evidence of harm (M) leading to refutation of healthcare professionals’ messages (O)
CMOC4	In services where midwives are enabled to deliver regular Carbon Monoxide (CO) monitoring alongside regular discussions of harm in a supportive, non-judgmental manner (C), women’s beliefs about harm can change overtime (M) leading to improved willingness to explore smoking cessation support (O)
***Promoting support***	
CMOC5	Where women have not disclosed smoking due to concern of being judged, and the routine nature of CO monitoring is not well explained by midwives (C) women can feel under surveillance, confirming their belief and expectation of being judged (M) leading to defensiveness, distrust, and disengagement (O)
CMOC6	Where midwives and the local stop smoking service (SSS) are well linked and there is clear understanding and communication about referral and the support available from the (SSS) (C) women feel assured and concerns about being judged are allayed (M) leading to interest and positivity to engage with the SSS (O) However, where there are poor links between services and referrals are not clearly communicated (C) women can feel unclear of what to expect, anxious and feel that choice has been taken away from them (M) leading to disempowerment, distrust, and lack of willingness to engage (O)
CMOC7	Where services have not implemented opt-out referrals, the criteria is unclear or midwives perceive women are not ready to be referred (C) midwives may use their professional judgement to decide when to refer, or adapt their communication about the process to make it more acceptable to women (M) leading to inconsistent implementation of referral pathways and failure to create a shared understanding with women of the importance and benefits of accessing support (O)
***Managing cravings***	
CMOC8	In organisations which do not receive or have confidence in the research about quit aids, or do not have access to appropriate training (C) healthcare professionals can feel unconfident and unwilling to advise and promote use (M) leading to women receiving incorrect or mixed messages, lack of confidence and unwillingness to use them (O)
CMOC9	Where women receive information about safety and acceptability of quit aids from those in their social network, the media or through public opinion (C) women consider these to be trusted, credible sources of information (M) leading to lack of confidence, unwillingness to use or decreased opportunities to use due to concern about being judged (O)
CMOC10	Where services provide quit aids that are insufficient dosage, without information and support of how to use or where women experience side effects (C) women can feel ashamed of struggling and try to adapt use to manage their cravings (M) leading to early termination of use, concern about nicotine levels, potential rationalisation of smoking being less harmful or relying on willpower to quit (O)
CMOC11	Where services provide a range of quit aids and offer support and flexibility to find the right type (C) women feel able to report struggling as they believe their experience is normal (M) leading to engagement with the service and willingness to try other options (O)
CMOC12	In services that make quit aids directly available and free of charge (C) women find them easier to access, concerns about affordability are relieved and are assured of their safety and acceptability (M) leading to improved willingness to use (O)
***Maintaining commitment***	
CMOC13	In services that offer financial incentives alongside behavioural support (C) women make a positive association with stopping smoking and plan and set their own goals (M) leading to frequent engagement with the service validate quit, increased opportunities to receive support and see their progress, improved self-efficacy and greater odds of stopping smoking (O)
CMOC14	Where healthcare professionals and/or women hear or perceive controversy or negative public opinion around financial incentives (C) healthcare professionals can feel uncomfortable about promoting the offer, and women can feel judged for smoking and guilty for being offered incentives (M) leading to unwillingness to engage with the service and reduced opportunities to do so (O)
CMOC15	Where women believe that smoking eases stress (C) women assess the perceived harm of stress on foetal health and development against the perceived harm of smoking and what other available coping strategies they have (M) leading to reduced motivation, and potential rationalisation of harm and choice to smoke (O)
CMOC16	Where services provide regular contact that is tailored to individual women’s needs and preferences (C) women feel understood and valued and face fewer physical barriers to engaging with the service (M) leading to regular opportunities to develop new coping strategies, to reinforce motivation and to build self-efficacy (O)
CMOC17	Where services offer support or incentives to a partner or supporter to stop smoking (C) awareness of harm and desire to support women can lead them to adapt their smoking to reduce prompts for women but may fail to motivate them to quit (M) leading to reduced exposure to second hand smoke, but also a sense of loss for women over shared activities and time together and tension and stress in relationships (O)
***Building self-efficacy***	
CMOC18	Where women experience pressure from their partner to stop smoking (C) women can feel judged, resentful and a loss of choice and control (M) leading to lack of motivation or self-efficacy to stop smoking and concern about creating tension and stress in their relationship (O)
CMOC19	Where healthcare professionals offer non-judgemental support (C) women feel understood and able to be honest (M) leading to greater engagement with the service, frequent opportunities to receive encouragement and support, improved self-efficacy and perseverance to quit (O) However, where women perceive or receive judgement from healthcare professionals (C) they feel judged, shame or defensive (M) leading to lowered self-efficacy and disengagement (O)

### Domain 1: Articulating harm

CMOCs 1—4 relate to how services build psychological capability by educating women about the harms of smoking in pregnancy to improve their capacity to stop smoking [8].

Midwives play a vital role in identifying smokers, initiating discussions about harm, and promoting smoking cessation. Several papers highlighted differences in the availability and uptake of smoking cessation training amongst midwives [3, 27, 31, 45, 50], and that training primarily focusses on understanding risks and delivering very brief advice [42]. This may cause midwives to feel ill equipped to discuss smoking cessation with some women [3, 27, 31, 45], resulting in inconsistent portrayals of harm [3, 27, 31, 34], and potential implied acceptance where women expect smoking to be discussed [3, 24]. Additionally, several papers underscored the tension for midwives between considering promoting smoking cessation to be important part of their role [3, 47] and the importance of maintaining a positive relationship with women [3, 24, 31]. Concerns about being perceived as judgemental [3, 24, 31] and knowledge of women’s existing views on smoking [24, 29] contribute to the professional judgements midwives make about if and how to discuss smoking. This can lead to missed opportunities to influence women’s views, or in diluting messages of harm to avoid distress or damaging the relationship therefore perpetuating women’s beliefs [31].

The way women evaluate midwives’ portrayal of harm can also be influenced by their contact with other messages and evidence of harm. Where women have smoked in a previous pregnancy or have those within their social network who have, the health of those children may be assessed as more tangible, realistic evidence of harm [3, 23, 24, 26, 31, 34]. However, several papers indicated that Carbon Monoxide (CO) monitoring, can be an effective way to communicate harm in a tangible way [3, 31–33, 51, 53]. Importantly, there is some evidence that where the risk of harm is discussed regularly in a supportive, non-judgmental manner, using CO monitoring, women’s beliefs about harm can change overtime, improving their willingness to explore smoking cessation support [3, 21, 31–34, 43, 51, 53].

### Domain 2: Promoting support

CMOCs 5—7 relate to how services build physical opportunity by providing services and support to enable behaviour change [8].

This analysis shows that the pathway from midwives identifying smokers to referral and take up of support from the local stop smoking service (SSS) can be a difficult but important transition. Concern about being judged was reported throughout many papers in this analysis [3, 21–24, 29, 33–35, 40]. This can cause women not to disclose smoking and therefore, if the routine nature of CO monitoring in antenatal appointments is not well communicated, its use maybe considered a surveillance tool. This may confirm women's concerns and beliefs of being judged, leading to distrust and disengagement [24, 33].

Several papers showed that where positive links between midwives and the SSS are made, this can be conducive to a clear referral process and clarity about the support the SSS can provide. This can offer assurance to women, helping to allay their concerns and create interest and positivity in engaging with the SSS [3, 21, 24, 32, 46]. Conversely, poor links between services can result in the referral and support being poorly communicated. This can fail to allay women’s concerns leading to distrust, disempowerment and lack of motivation to engage [3, 21–24, 29, 31–33]. Despite recommendations for all women who smoke to receive referral for support [15], not all do. Inconsistencies can occur where services have not implemented opt-out referrals, the criteria are unclear or midwives perceive that women are not ready to be referred [3, 21, 31, 33]. This can result in professional judgement being used to decide when to refer, or, adapting communication about the referral process to make it appear more acceptable. This can result in failure to promote the importance and benefits of engaging with the SSS [3, 21, 31, 33].

### Domain 3: Managing cravings

CMOCs 8—12 relate to how services build psychological capability by educating women about quit aids (nicotine replacement therapy and vapes/e-cigarettes), physical capability to improve their stamina to manage nicotine cravings and physical opportunity by providing quit aids to enable behaviour change [8].

This analysis shows knowledge and confidence about the safety and effectiveness of quit aids is varied across healthcare professionals. The availability and quality of research, dissemination to and within organisations [27, 30] and training [3, 27, 31, 42, 50] can impact confidence and willingness to advise and promote use. Whilst training about quit aids is central for SSS advisors [50], its place in midwives training is less prevalent, therefore reducing their capability to advise [42]. This can lead to women receiving incorrect or mixed messages about safety resulting in unwillingness to use [3, 25, 26, 28, 30, 31, 36, 37, 44]. Although women’s confidence of products is highly influenced by the recommendation of healthcare professionals [44], it is also influenced by other messages of safety. Information and stories shared by their social network, the media [25, 26, 28, 30, 41, 44] or the perceived public opinion of the acceptability of vapes [28, 29, 35, 37, 41] can reduce women’s willingness to use [36] or their opportunities to use due to concerns about judgement [28, 29, 35, 37, 41].

Additionally, previous experience of quit aids was reported across several papers as an influencing factor to women's perception of harm and use. Insufficient dosage [23, 25, 28, 35], side effects [28, 30] and lack of information about how to use the products [23] can cause women to struggle to manage cravings leading to frustration, shame, and trying to adapt product use to improve its effectiveness. This can lead to discontinuation, concerns about the level of nicotine being consumed, potential rationalisation of smoking being less harmful or relying on willpower alone [25, 26, 28, 30, 37]. However, there is some evidence that where services provide a range of quit aids with flexibility and support to try different types, struggling to manage cravings may be ‘normalised’ leading to engagement with the service and willingness to try other types [22, 30]. Finally, several papers indicated that where services make quit aids directly available and free of charge, this can provide easier access and act as an endorsement of safety and acceptability, leading to improved willingness to use [3, 21, 22, 25, 28].

### Domain 4: Maintaining commitment.

CMOCs 13 – 17 relate to how services build motivation through planned behaviour and managing habitual processes and emotional responses, and through social opportunity by changing the social cues associated with smoking [8].

Several papers highlighted that financial incentives improve ongoing engagement with the SSS and can enable a successful quit. Financial incentives can promote a positive association with stopping smoking [22, 40] causing women to make plans and set their own goals for how to use the money [38]. Frequent engagement with the service to validate the smoking quit and receive incentives also provides frequent encouragement and visual proof of women’s achievements. This contributes to improved self-efficacy [3, 22, 38–40] and improved odds of smoking cessation [48, 49]. However, engagement with services offering financial incentives may be influenced by public opinion. Heard or perceived controversy or negative public opinion about financial incentives them can cause advisors to feel uncomfortable and women to feel judged. This can result in unwillingness to participate or reduced opportunities to do so [3, 21, 22].

Support from services to maintain resolve to not smoke can be influenced by women’s belief that smoking relieves stress. Stress can cause motivation to waver, leading to an assessment of other coping strategies and the perceived risk of smoking compared to stress [22, 25, 34, 44, 46]. However, several papers indicated that services that make regular contact and provide tailored support can bolster women’s motivation, and self-efficacy. Making women feel understood and valued, removing physical barriers to engaging with the service can lead to opportunities to develop new coping strategies, reinforce motivation, and build self-efficacy [3, 22, 31, 34, 46].

Finally, some papers show that where services offer support or incentives to partners or supporters to stop smoking, this is generally not well taken up [3, 22]. Desire to support women may result in willingness to adapt their smoking to reduce prompts and exposure to second hand smoke but may fail to motivate them to quit. This can cause a sense of loss over shared activities and time together and cause tension and stress in relationships where differences in smoking underscores the responsibility for foetal health placed on women [3, 22].

### Domain 5: Build self-efficacy

CMOCs 18—19 relate to how services build psychological capability by building women’s capacity to continue to engage in the process of smoking cessation and reflective motivation through analysing progress and circumstances to plan for behaviour change [8].

Pressure from a partner to stop smoking can cause women to feel judged and that choice and control is being taken away from them. This can lead women to lack motivation or self-efficacy to stop smoking, expressed through defiance, disempowerment and hiding their smoking [3, 29, 35]. However, several papers highlighted that the way healthcare professionals discuss smoking and promote support are important to women’s beliefs about their power and capability to stop smoking. Non-judgemental support can make women feel understood leading them to form trusted relationships with healthcare professionals where they can be honest about challenges and receive regular encouragement and support to see their achievements. This can lead to improved self-efficacy and perseverance to quit. Conversely, where women perceive judgement from healthcare professionals, they may feel judged and ashamed leading to lowered self-efficacy and disengagement [3, 22–24, 29, 34, 47].

## Discussion

### Summary of findings

The aim of this review was to improve the understanding of how services in the UK to reduce smoking in pregnancy work, for whom, and under what circumstances. The review resulted in an explanatory model, structured over five interconnected domains that provides clarity of how services work, the contexts in which behaviour change mechanisms are tiggered, or not, and an understanding of how and why outcomes vary. The ability of services to achieve their intended outcomes is strengthened by aligning efforts across all five domains. However, this interconnectedness can also initiate reinforcing relationships which can reduce a services’ ability to trigger behaviour change mechanisms.

This review identifies two key processes involved in how services achieve their effects: how material resources are implemented and relationships. CO monitoring that is well explained and delivered regularly can improve women’s psychological capability about harm. Clearly communicated opt-out referrals that promote the benefits and importance of engaging with the service can improve the physical opportunities women have to support them. Easy access to quit aids, where flexibility is offered to find the right type, can improve women’s physical capability by managing nicotine cravings to improve stamina and reduce the automatic motivation to smoke when faced with cravings or stress. Financial incentives that are well promoted and delivered alongside individualised behavioural support can build women’s reflective motivation through making plans and setting goals as well as regularly seeing their progress and achievements. Whilst the use of material resources produces some understanding of how services can create conditions that trigger behaviour change mechanisms, it risks reducing the understanding of behaviour change to a set of rationale and logical decisions based on the information and opportunities presented [9]. Many of the papers included in this review reflect that how services implement material resources alone does not produce a consistent and predictable set of outcomes.

This review has also found that the relationships between women and healthcare professionals are vital to creating the conditions in which behaviour change can occur and how the implementation of material resources can have their intended effects. Non-judgemental support, regular contact and encouragement make women feel important, cared for, and can build women’s self-efficacy to face the challenges of smoking cessation. Importantly, the development of a positive relationship can place healthcare professionals as a trusted advisor and ally for women. Some papers included in this review report that women found that the support of healthcare professionals made them feel they were not alone and that they felt accountable to the advisor [3, 22]. This suggests that women’s relationship with healthcare professionals can play an important role in interrupting the social cues and social practice of smoking, even where those around the woman continue to smoke.

### Comparison with existing literature

The findings of this review are consistent with the findings and conclusion of the HTA undertaken in 2017 [3] which identified women’s smoking related perceptions and experiences to be fluid and context dependent. The majority of the papers (*n* = 21) included in this review were published from 2018 onwards, after the HTA was published, therefore reflecting the continued complexities in how services are experienced by women. The explanatory model developed by this review contributes to the understanding of how services can take account of the interplay between individual, interpersonal and environmental aspects of women’s lives and seek to operate at these different levels simultaneously.

### Strengths and limitations

A strength of this review is that it has brought together existing literature across a range of interventions and approaches, often studied in isolation. A further strength is the range of available study designs included, particularly the large amount of qualitative research which provided a good level of reporting of contexts and mechanisms. However, a limitation is the variable amount and quality of research undertaken across the different interventions and approaches. The programme theory developed offers limited explanatory insights on how tailored behavioural support to women and their partners or supporters are delivered, how they work and to what outcomes. Existing literature was considerably less rich and well explored in this areas compared to CO monitoring, opt-out referrals, quit aids and financial incentives. Whilst behavioural support has been recognised as an important component to achieving smoking cessation in the short term [3], specific behaviour change techniques, if used, were not reported. This presents limitations in the understanding of how these techniques may or may not be helpful in different contexts. Rather, reporting focussed on the venues used for service delivery, the methods of contact and how these may contribute to creating opportunities that enable women’s continued engagement with services.

The process undertaken to assess the relevance and rigour of included studies is a strength of the approach taken, providing assurance of the quality of the studies included in this review, and of the programme theory generated from them. However, a potential weakness of the approach may be the search terms used. Whilst these were tested and developed iteratively, they could have been more comprehensive to cover the breadth of sources relating to the topic of interest, and therefore it is possible that relevant studies were missed. As with all realist approaches, the programme theory generated is considered to always be in development [10], and therefore, the next stage would be to review and iterate the model based on new evidence as it emerges.

## Conclusions

This review clarifies the range of interconnected and bi-directional relationships between services and the personal and social factors of women’s lives. It underscores the importance of aligning efforts across the models five domains to strengthen services’ ability to trigger behaviour change mechanisms to achieve smoking cessation. The review identifies two key processes involved in how services achieve their effects: how material resources are implemented and relationships.

### Recommendations for policy and practice

This review highlights the need for improved communication about the safety and effectiveness of quit aids, specifically vapes, and of the benefits of financial incentives. Recent advancements in research in these areas [48, 49, 54] show they can positively affect behaviour change. Therefore, clear, and consistent messages through policy are vital to improving healthcare professionals’ knowledge and to endorse acceptability both to professionals and members of the public.

Acknowledging the range of different service configurations across the UK, commissioners and service providers are recommended to use this review to explore their service provision against the five domains in the explanatory model to identify areas for development relevant to them. However, an important finding of this review has been the importance of relationships, not just of how material resources are implemented. Therefore, services are recommended to identify ways in which positive relationships can be built. This may include identifying how services can facilitate more regular contact with pregnant women and by providing training and development in coaching and motivational interviewing techniques to enhance the skills of healthcare professionals to facilitate behaviour change.

### Further research

Future research should focus on improving the understanding of the relationships between women, their social networks and healthcare professionals in relation to how smoking is maintained and how behaviour is changed. Existing literature in this review found heterogeneity in whether the smoking behaviours of those closest to women affected their capability and motivation to stop smoking, but the way differences occurred was not consistently explored or understood. Longitudinal approaches may support an understanding of how relationships influence beliefs and behaviours overtime.

### Supplementary Information

**Additional file 1. **Search strategy and terms.

**Additional file 2. **Appraisal judgements on included studies.

## Data Availability

All data generated or analysed during this study are included in this published article [and its supplementary information files].

## Abbreviations

ASH: Action on Smoking and Health
CASP: Critical appraisal skills programme
CMOC: Context mechanism outcome configuration
CO monitoring: Carbon monoxide monitoring
HTA: Health Technology Assessment
NICE: National Institute for Health and Care Excellence
NRT: Nicotine replacement therapy
NCSCT: National Centre for Smoking Cession and Training
Quit aids: Nicotine replacement therapy including vapes/e-cigarettes
SATOD: Smoking at time of delivery
SSS: Stop Smoking Service
