# Supplementary material for: Understanding for whom, under what conditions and how smoking cessation services for pregnant women in the United Kingdom work—a rapid realist review
Source: BMC Public Health. 2023 Dec 12;23:2488. doi: 10.1186/s12889-023-17378-w (PMC10717267; doi:10.1186/s12889-023-17378-w)
Supplement: Supplementary file 1 — Additional file 1. Search strategy and terms. [file 12889_2023_17378_MOESM1_ESM.docx]

Additional file 1 – search strategy and terms

Databases: CAB Abstracts, Embase, Global Health, OVID MEDLINE(R),

Host: Ovid

1. (pregnan*.ti,ab OR maternal.ti,ab)
2. (smoking.ti,ab OR tobacco.ti,ab OR cigarette.ti,ab)
3. (quit.ti,ab OR cessation.ti,ab OR reduction.ti,ab OR abstinence.ti,ab OR stop.ti,ab)
4. (United Kingdom.ti,ab OR UK.ti,ab)

1 AND 2 AND 3 AND 4
