# Supplementary material for: Understanding for whom, under what conditions and how smoking cessation services for pregnant women in the United Kingdom work—a rapid realist review
Source: BMC Public Health. 2023 Dec 12;23:2488. doi: 10.1186/s12889-023-17378-w (PMC10717267; doi:10.1186/s12889-023-17378-w)
Supplement: Supplementary file 2 — Additional file 2. Appraisal judgements on included studies. [file 12889_2023_17378_MOESM2_ESM.docx]

Additional file 2 – appraisal judgements on included studies

***Relevance rating criteria:***

High relevance – paper offers a significant contribution to theory building based on strength of contribution across all three criteria.

Somewhat relevant – paper offers some contribution to theory building, lacking strength of evidence against some of the three criteria.

Limited relevance – paper offers a little contribution to theory building, being unable to contribute across all three criteria.

***Rigor rating criteria:***

High – paper provides evidence and assurance across all three criteria.

Medium – paper provides evidence and assurance across the majority of the three criteria.

Low - paper provides evidence and assurance in only one aspect of the criteria.

**Table 1 – study level relevance and rigour ratings**

| **Title** | Is the intervention studied similar to the initial program theory or of other search results? | Does the study contribute to an understanding of the different level(s) the service operated at or was experienced at? | Does the study contribute to an understanding of the mechanisms that the service intended to trigger or that were observed? | Relevance rating | Is the study design appropriate to the research question/aims? | Is there evidence of methodological rigor? | Is there evidence of critical analysis of the findings? | Rigor rating |
| --- | --- | --- | --- | --- | --- | --- | --- | --- |
| **McKell. J et al.** (2022) *Usual care in a multicentre randomised controlled trial of financial incentives for smoking cessation in pregnancy: qualitative findings from a mixed methods process evaluation*.  <http://dx.doi.org/10.1136/bmjopen-2022-066494> | Yes | Yes | Yes | High | Yes | Yes | Yes | High |
| **McCormack F.C et al.** (2022) *Exploring pregnant women's experiences of stopping smoking with an incentive scheme with 'enhanced' support: a qualitative study.*  <https://doi.org/10.1177/17579139221106842> | Yes | Yes | Yes | High | Yes | Yes | Yes | High |
| **Stacey T. et al.** (2022) *'I don't need you to criticise me, I need you to support me'. A qualitative study of women's experiences of and attitudes to smoking cessation during pregnancy.*  <https://doi.org/10.1016/j.wombi.2022.01.010> | Somewhat | Yes | Somewhat | Somewhat | Yes | Yes | Yes | High |
| **Griffiths S.E et al.** (2021) *Accessing specialist support to stop smoking in pregnancy: A qualitative study exploring engagement with UK-based stop smoking services.*  <https://doi.org/10.1111/bjhp.12574> | Yes | Yes | Yes | High | Yes | Yes | Yes | High |
| **McDaid L. et al.**  (2020) *Understanding pregnant women's adherence-related beliefs about Nicotine Replacement Therapy for smoking cessation: A qualitative study.* <https://doi.org/10.1111/bjhp.12463> | Yes | Yes | Yes | High | Yes | Yes | Yes | High |
| **R Froggatt S. et al.** (2021)  *Risk perception of cigarette and e-cigarette use during pregnancy: A qualitative postpartum perspective.*  <https://doi.org/10.1016/j.midw.2020.102917> | Yes | Yes | Somewhat | Somewhat | Yes | No | Yes | Medium |
| **Hunter A. et al.**  (2020) *Healthcare Professionals' Beliefs, Attitudes, Knowledge, and Behaviour around Vaping in Pregnancy and Postpartum: A Qualitative Study.*  <https://doi.org/10.1093/ntr/ntaa126> | Yes | Yes | Yes | High | Yes | Yes | No | Medium |
| **Campbell K. et al.** (2020) *Factors influencing the uptake and use of nicotine replacement therapy and e-cigarettes in pregnant women who smoke: a qualitative evidence synthesis.*  <https://doi.org/10.1002/14651858.CD013629> | Yes | Yes | Yes | High | Yes | Yes | Yes | High |
| **Grant A. et al.** (2020) *Smoking during pregnancy, stigma and secrets: Visual methods exploration in the UK.*  <https://doi.org/10.1016/j.wombi.2018.11.012> | Yes | Yes | Yes | High | Yes | Yes | Yes | High |
| **Thomson R. et al.**  (2019) *Knowledge and education as barriers and facilitators to nicotine replacement therapy use for smoking cessation in pregnancy: A qualitative study with health care professionals.*  <https://doi.org/10.3390/ijerph16101814> | Yes | Yes | Yes | High | Yes | Yes | Yes | High |
| **Naughton F. et al.** (2018) *Barriers and facilitators to smoking cessation in pregnancy and in the post-partum period: The health care professionals' perspective.*  <https://doi.org/10.1111/bjhp.12314> | Yes | Yes | Yes | High | Yes | Yes | Yes | High |
| **Campbell K.A. et al.** (2016) *Antenatal clinic and stop smoking services staff views on "opt-out" referrals for smoking cessation in pregnancy: A framework analysis.*  <https://doi.org/10.3390/ijerph13101004> | Yes | Somewhat | Yes | Somewhat | Yes | No | Yes | Medium |
| **Sloan M. et al.** (2016) *Pregnant Women's Experiences and Views on an "Opt-Out" Referral Pathway to Specialist Smoking Cessation Support: A Qualitative Evaluation.*  <https://doi.org/10.1093/ntr/ntv273> | Yes | Yes | Yes | High | Yes | Yes | Yes | High |
| **Bauld L. et al.** (2017)  *Barriers to and facilitators of smoking cessation in pregnancy and following childbirth: Literature review and qualitative study.*  <https://doi.org/10.3310/hta21360> | Yes | Yes | Yes | High | Yes | Yes | Yes | High |
| **Bluegrass.** (2022)  *Action on Smoking and Health: Qualitative insights.*  <https://ash.org.uk/uploads/Qualitative_Insights_Primary_Research_Report_2022-12-02-140553_trte.pdf?v=1669989950> | Yes | Somewhat | Yes | Somewhat | Yes | No | Yes | Medium |
| **Grant. A. et al.** (2019) *Understanding health behaviour in pregnancy and infant feeding intentions in low-income women from the UK through qualitative visual methods and application to the COM-B model.* <https://doi.org/10.1186/s12884-018-2156-8> | Somewhat | Somewhat | Somewhat | Somewhat | Yes | Yes | Yes | High |
| **Cooper S. et al.** (2019) *Attitudes to E-cigarettes and cessation support for pregnant women from English stop smoking services: A mixed methods study.* <https://doi.org/10.3390/ijerph16010110> | Yes | Yes | Somewhat | Somewhat | Yes | Yes | Yes | High |
| **Bowker K. et al.** (2018) *Views on and experiences of electronic cigarettes: A qualitative study of women who are pregnant or have recently given birth.* <https://doi.org/10.1186/s12884-018-1856-4> | Yes | Somewhat | Somewhat | Somewhat | Yes | Yes | Yes | High |
| **Crossland N. et al.** (2015) *Incentives for breastfeeding and for smoking cessation in pregnancy: An exploration of types and meanings.* <https://doi.org/10.1016/j.socscimed.2014.12.019> | Somewhat | No | Somewhat | Limited | Yes | Yes | No | Medium |
| **Thomson G. et al.** (2014) *Unintended consequences of incentive provision for behaviour change and maintenance around childbirth.* <https://doi.org/10.1371/journal.pone.0111322> | Somewhat | Somewhat | No | Limited | Yes | Yes | Yes | High |
| **Mantzari E. et al.** (2012) *The effectiveness of financial incentives for smoking cessation during pregnancy: Is it from being paid or from the extra aid?* <https://doi.org/10.1186/1471-2393-12-24> | Yes | Somewhat | Yes | Somewhat | Yes | Yes | Yes | High |
| **Moyse M. et al.** (2020) Newspaper *media representation of electronic cigarette use during pregnancy.* <https://doi.org/10.1093/pubmed/fdaa048> | Somewhat | Somewhat | No | Limited | Yes | No | Yes | Medium |
| **Forman J. et al.** (2017)  *National survey of smoking and smoking cessation education within UK Midwifery School curricula.* <https://doi.org/10.1093/ntr/ntw230> | Yes | Somewhat | No | Somewhat | Yes | No | Yes | Medium |
| **O'Connell M & Duaso M**  (2014) *Barriers and facilitators of midwives' use of the carbon monoxide breath test for smoking cessation in practice: a qualitative study.*  <https://www.researchgate.net/publication/269699226_Barriers_and_facilitators_of_midwives'_use_of_the_carbon_monoxide_breath_test_for_smoking_cessation_in_practice_a_qualitative_study> | Yes | Somewhat | No | Limited | Yes | No | No | Low |
| **Bowker. K et al.** (2020)  *Pregnant women's use of e-cigarettes in the UK: a cross-sectional survey*. <https://doi.org/10.1111/1471-0528.16553> | Yes | Somewhat | No | Limited | Yes | Yes | Yes | High |
| **Naughton F. et al.** (2019) *Interest in and Use of Smoking Cessation Support across Pregnancy and Postpartum.* <https://doi.org/10.1093/ntr/ntz151> | Yes | Somewhat | Somewhat | Somewhat | Yes | Yes | Yes | High |
| **Vaz L.R. et al.** (2017)  Factors associated with the effectiveness and reach of NHS stop smoking services for pregnant women in England. <https://doi.org/10.1186/s12913-017-2502-y> | Yes | Somewhat | No | Limited | Yes | Yes | Yes | High |
| **Campbell K.A. et al.** (2016) *'Opt-out' referrals after identifying pregnant smokers using exhaled air carbon monoxide: impact on engagement with smoking cessation support.* <http://dx.doi.org/10.1136/tobaccocontrol-2015-052662> | Yes | No | Somewhat | Limited | Yes | Yes | Yes | High |
| **Beenstock J. et al.** (2012)  *What helps and hinders midwives in engaging with pregnant women about stopping smoking? A cross-sectional survey of perceived implementation difficulties among midwives in the North East of England.* <https://doi.org/10.1186/1748-5908-7-36> | Yes | Somewhat | No | Limited | Yes | Yes | Yes | High |
| **Tappin D. et al.** (2022)  *Effect of financial voucher incentives provided with UK stop smoking services on the cessation of smoking in pregnant women (CPIT III): Pragmatic, multicentre, single blinded, phase 3, randomised controlled trial.* <https://doi.org/10.1136/bmj-2022-071522> | Yes | No | Somewhat | Limited | Yes | Yes | Yes | High |
| **Tappin D. et al.** (2015) *Financial incentives for smoking cessation in pregnancy: Randomised controlled trial.* <https://doi.org/10.1136/bmj.h134> | Yes | No | Somewhat | Limited | Yes | Yes | Yes | High |
| **Thomson R. et al.** (2022)  *Smoking Cessation Support for Pregnant Women Provided by English Stop Smoking Services and National Health Service Trusts: A Survey.* <https://doi.org/10.3390/ijerph19031634> | Yes | Somewhat | No | Limited | Yes | No | Yes | Medium |
| **Local Government Association** (2018) *Fit for and during pregnancy: a key role for local government.*  <https://www.local.gov.uk/sites/default/files/documents/15.52%20Fit%20for%20and%20during%20pregnancy_03.pdf> | Yes | No | Somewhat | Limited | Yes | No | No | Low |

**Table 2 – CASP checklist responses (qualitative studies)**

| **Study** | Was there a clear statement of the aims of the research | Is a qualitative methodology appropriate? | Was the research design appropriate to address the aims of the research? | Was the recruitment strategy appropriate to the aims of the research? | Was the data collected in a way that addressed the research issue? | Has the relationship between researcher and participants been adequately considered? | Have ethical issues been taken into consideration? | Was the data analysis sufficiently rigorous? | Is there a clear statement of findings? |
| --- | --- | --- | --- | --- | --- | --- | --- | --- | --- |
| **McKell. J et al.** (2022) *Usual care in a multicentre randomised controlled trial of financial incentives for smoking cessation in pregnancy: qualitative findings from a mixed methods process evaluation*. <http://dx.doi.org/10.1136/bmjopen-2022-066494> | Yes | Yes | Yes | Yes – study included diverse range of participants including staff and pregnant women | Yes – design comprised site observations and in-depth, semi-structured interviews | Yes – interviewers had experience of research in smoking cessation and pregnancy | Yes – ethics approval granted as part of the wider RCT | Yes – however, detail on how realist principles were applied and how CMOCs were derived was limited | Yes – summary of findings and comparison to existing literature |
| **McCormack F.C et al.** (2022) *Exploring pregnant women's experiences of stopping smoking with an incentive scheme with 'enhanced' support: a qualitative study.*  <https://doi.org/10.1177/17579139221106842> | Yes | Yes | Yes – inclusion of pregnant smokers and advisors offers broader range of views. However, sample size was small | Yes - however, whilst identification of women through advisors potentially helped recruitment, it may have led to bias in the selection of women and therefore reduced breadth of views and experiences | Yes | Yes – researchers had appropriate experience and women were made aware of the researchers position and involvement in the scheme | Yes – ethics approval was received, and consent was sought for involvement | Yes – analysis strategy was discussed including roles of researchers involved | Yes – key themes clearly identified and reflection of these themes in the context of existing research provided. |
| **Stacey T. et al.** (2022) *'I don't need you to criticise me, I need you to support me'. A qualitative study of women's experiences of and attitudes to smoking cessation during pregnancy.* <https://doi.org/10.1016/j.wombi.2022.01.010> | Yes | Yes | Yes – interview guide provided | Yes – recruitment undertaken by social media may have engaged a broader range of women than recruiting from stop smoking services | Yes | Yes – however, researchers were midwives. Potential reflexivity from their position was explored. | Yes – ethics approval was received, and consent was sought for involvement | Yes – thematic analysis and agreement between several researchers | Yes – summary of findings and comparison to existing literature |
| **Griffiths S.E et al.** (2021) *Accessing specialist support to stop smoking in pregnancy: A qualitative study exploring engagement with UK-based stop smoking services.*  <https://doi.org/10.1111/bjhp.12574> | Yes – although the aims were quite broad. | Yes | Yes – inclusion of pregnant smokers and advisors offers broader range of views. | Yes – recruitment undertaken both by midwives and stop smoking advisers, and via social media. | Yes – clear explanation of interview process and data saturation | Yes – interviewers had experience of research in smoking cessation and pregnancy | Yes – ethics approval received, permission from NHS Trusts received and individual consent sought | Yes – use of theoretical domain framework and COM-B system of behaviour change to analyse data and identify themes. | Yes – summary of what is already known and what this research adds |
| **McDaid L. et al. (**2020) *Understanding pregnant women's adherence-related beliefs about Nicotine Replacement Therapy for smoking cessation: A qualitative study.* <https://doi.org/10.1111/bjhp.12463> | Yes – focus on experience with NRT to understand low uptake | Yes | Yes – however number of participants was small | Yes – recruitment undertaken across multiple channels. Approach noted as having achieved good representation of those of low socioeconomic position | Yes – details of how emergent themes through interviews were handled. | Yes – interviewer had experience in qualitative research. However, unclear of any prior experience with smoking cessation and/or pregnancy. | Yes – ethics approval received, permission and individual consent sought | Yes – data analysis guided by Necessity-Concerns Framework to draw themes and conclusions | Yes – summary of what is already known and what this research adds |
| **R Froggatt S. et al.** (2021)  *Risk perception of cigarette and e-cigarette use during pregnancy: A qualitative postpartum perspective.*  <https://doi.org/10.1016/j.midw.2020.102917> | Yes – focus on e-cigarette use and perceptions | Yes | Yes – interview questions provided. | Yes – recruitment taken from another, larger study with details reported. | Yes – questions based on literature review and data collection methods reported. | No – researcher and their position not discussed | Yes – ethics approval received; however individual consent not discussed | Can’t tell – limited details reported | Yes – summary of findings provided alongside discussion of breadth of research in this area and contradictions in existing evidence |
| **Hunter A. et al.**  (2020) *Healthcare Professionals' Beliefs, Attitudes, Knowledge, and Behaviour around Vaping in Pregnancy and Postpartum: A Qualitative Study.*  <https://doi.org/10.1093/ntr/ntaa126> | Yes – focus on e-cigarette use and perceptions | Yes | Yes – good sample size and range of healthcare professionals | Yes – range of methods and efforts used to engage a range of healthcare professionals recruited | Yes – topic guide provided and discussion of data saturation. | No – researcher and their position not discussed | Yes – ethics approval received, permission and individual consent sought | Yes – use of theoretical domain framework and COM-B system of behaviour change to analyse data and identify themes. | Yes – summary of findings provided alongside discussion of breadth of research in this area and recommendations for policy and practice |
| **Grant A. et al.** (2020) *Smoking during pregnancy, stigma and secrets: Visual methods exploration in the UK.*  <https://doi.org/10.1016/j.wombi.2018.11.012> | Yes – aim to focus on socioecological context of smoking | Yes | Yes – small number of participants, However, design produced depth of responses and insights | Yes – range of methods used but targeted to achieve recruitment of the intended participants | Yes | Yes – discussion on approaches taken to address power imbalances between participants and researcher | Can’t tell – ethics procedure not clearly reported. Individual participant consent sought. | Yes | Yes |
| **Thomson R. et al.**  (2019) *Knowledge and education as barriers and facilitators to nicotine replacement therapy use for smoking cessation in pregnancy: A qualitative study with health care professionals.*  <https://doi.org/10.3390/ijerph16101814> | Yes – focussed to NRT adherence from the healthcare professional perspective | Yes | Yes – good sample size and range of healthcare professionals | Can’t tell – methods not reported | Yes – topic guide provided which had been trialled in previous, similar research to stimulate conversation about the research area of interest | Yes – researchers had appropriate experience and training with facilitating focus groups | Yes – ethics approval gained, and individual consent sought | Yes – process reported, and analysis framework was developed for the research to identify themes and sub-themes | Yes – findings clearly reported. However, limited reflection on how this research sits within the context of existing literature in the field. |
| **Naughton F. et al.** (2018) *Barriers and facilitators to smoking cessation in pregnancy and in the post-partum period: The health care professionals' perspective.*  <https://doi.org/10.1111/bjhp.12314> | Yes | Yes | Yes – interview topic guide provided. Focus group used to test and develop further findings of semi-structured interviews | Yes – quota was determined in advance and met. Range of healthcare professionals approached | Yes – however, data saturation wasn’t reported | Yes - description of researcher's positions, training and experience in conducting interviews and focus groups provided. | No – ethics not reported. However, individual consent was sought | Yes – socioecological framework used, and process undertaken clearly reported | Yes – summary provided of what is already known and what this study adds |
| **Campbell K.A. et al.** (2016) *Antenatal clinic and stop smoking services staff views on "opt-out" referrals for smoking cessation in pregnancy: A framework analysis.*  <https://doi.org/10.3390/ijerph13101004> | Yes | Yes | Yes – interview topic guide provided | Yes – participants recruited from the NHS Trusts that wider service evaluation work was being conducted in | Yes | Yes – description of researcher's positions, and training provided. Researchers had an existing relationship with participants | Yes – categorised as service improvement rather than research. Therefore, permission from NHS trusts gained as opposed to ethics approval. Individual consent sought. | Yes – framework analysis used, and process undertaken clearly reported | Yes – findings clearly reported, including how viewpoints changed overtime and in the context of existing research |
| **Sloan M. et al.** (2016) *Pregnant Women's Experiences and Views on an "Opt-Out" Referral Pathway to Specialist Smoking Cessation Support: A Qualitative Evaluation.*  <https://doi.org/10.1093/ntr/ntv273> | Yes | Yes | Can’t tell – minimal information reported about the types of questions asked and approaches used. | Yes – recruitment undertaken from participants enrolled in a wider service evaluation | Yes | Can’t tell – minimal information reported | No – ethics not reported. However, individual consent sought | Yes – stages of the analysis clearly described | Yes – key themes reported, and findings reported in the context of existing research in this area |
| **Grant. A. et al.** (2019) *Understanding health behaviour in pregnancy and infant feeding intentions in low-income women from the UK through qualitative visual methods and application to the COM-B model.* <https://doi.org/10.1186/s12884-018-2156-8> | Yes | Yes | Yes – however, depth of methods used meant that only a small number of participants were involved | Yes – recruitment undertaken external to healthcare services. Characteristics of participants reported | Yes | Yes – clearly considered and reported | Yes – ethical approval gained, and individual consent sought | Yes – process thoroughly reported including coding and use of COM-B as analytical framework | Yes – however, findings were not clearly set out in the context of existing literature |
| **Bowker K. et al.** (2018) *Views on and experiences of electronic cigarettes: A qualitative study of women who are pregnant or have recently given birth.* <https://doi.org/10.1186/s12884-018-1856-4> | Yes – focus on electronic use of cigarettes | Yes | Yes – topic guide for semi structured interviews provided | Yes – breadth of avenues used to recruit different women. Both pregnant and postpartum women included. | Yes | Can’t tell – minimal information about the researchers provided and their relationship with participants | Yes – ethics approval provided, and individual consent sought | Yes – framework used, and process thoroughly reported | Yes – findings reported as key themes including comparison to existing literature |
| **Mantzari E. et al.** (2012) *The effectiveness of financial incentives for smoking cessation during pregnancy: Is it from being paid or from the extra aid?* <https://doi.org/10.1186/1471-2393-12-24> | Yes – focussed to understanding mechanisms by which financial incentives are effective | Yes | Yes – however, details of the questions or topic guide used were not clearly reported | Yes – participants were recruited from a trial involving financial incentives. Compare and contrast of viewpoints offered between those who received financial incentives and those who didn’t | Yes – flexibility of times and locations for interviews. Methods of data collection also reported | Can’t tell – limited information about the researcher reported | Yes – ethics approval received, and individual consent sought | Yes – analytical framework used. However, details or who and how analysis was undertaken was limited | Yes – however, findings were not well discussed in the context of existing literature. |
| **Crossland N. et al.** (2015) *Incentives for breastfeeding and for smoking cessation in pregnancy: An exploration of types and meanings.* <https://doi.org/10.1016/j.socscimed.2014.12.019> | Yes – however, focus of study may have benefitted from definitions of incentives | Yes | Yes – topic guide for interviews derived from evidence synthesis. Both interviews and semi-structured interviews used | Yes – participants were recruited across three geographic areas with different characteristics to achieve diversity within the sample | Yes | Yes – accounting for researcher reflexivity included in study design | Yes – ethics approval received, and individual consent sought | Yes – analytical framework used. | Yes – findings reported alongside the findings of an evidence synthesis |
| **O'Connell M & Duaso M**  (2014) *Barriers and facilitators of midwives' use of the carbon monoxide breath test for smoking cessation in practice: a qualitative study.*  <https://www.researchgate.net/publication/269699226_Barriers_and_facilitators_of_midwives'_use_of_the_carbon_monoxide_breath_test_for_smoking_cessation_in_practice_a_qualitative_study> | Yes – experiences of midwives from the initial implementation of CO monitoring as standard | Yes | Can’t tell – semi structured interviews conducted but no detail reported on questions used. | Can’t tell – minimal reporting. Whilst large pool was approached, only a small number of participants responded, mainly from one geographic area. Unclear why this may have been or what efforts were made to improve sampling. | Can’t tell – length of interviews and data saturation not discussed. | Can’t tell – limited information reported about the role of the researcher, their experience and training. | Yes – ethics approval granted. | Can’t tell – limited reporting on how the analysis was conducted. However, findings presented demonstrate a balance of both positive and negative viewpoints | Yes – findings well summarised in the context of limited research in this area. Conclusions drawn are clear and logical. |
| **Bauld L. et al.** (2017) *Barriers to and facilitators of smoking cessation in pregnancy and following childbirth: Literature review and qualitative study.* <https://doi.org/10.3310/hta21360> | Yes | Yes | Yes – perspectives sought from pregnant women, significant others and range of healthcare professionals to triangulate findings. Topic guide developed and used. | Yes – sampling strategy set out in advance to ensure appropriate diversity for the scope of the study | Yes – interview format and duration reported as well as the target interviews with different participants and what was actually conducted. | Yes – descriptions of interviewers, their experience and training provided. | Yes – ethics approval granted, and individual participant consent sought | Yes – use of socioecological framework for analysis | Yes |

**Table 3 – CASP checklist responses (randomised controlled trials)**

| **Study** | Did the study address a clearly focused research question? | Was the assignment of participants to interventions randomised? | Were all participants who entered the study accounted for at its conclusion? | Was blinding used? | Were the study groups similar at the start of the randomised controlled trial? | Apart from the experimental intervention, did each study group receive the same level of care? | Were the effects of intervention reported comprehensively? | Was the precision of the estimate of the intervention or treatment effect reported? | Do the benefits of the experimental intervention outweigh the harms and costs? |
| --- | --- | --- | --- | --- | --- | --- | --- | --- | --- |
| **Tappin D. et al.** (2022) *Effect of financial voucher incentives provided with UK stop smoking services on the cessation of smoking in pregnant women (CPIT III): Pragmatic, multicentre, single blinded, phase 3, randomised controlled trial.* <https://doi.org/10.1136/bmj-2022-071522> | Yes | Yes | Yes - flow diagram provided. Study benefitted from very low dropout rate following random assignment | Yes -  Call centre operatives gaining consent were blinded. However, participants were not blinded to their allocation. The impact of this was explored by the authors. | Yes | Can’t tell – trial recruited from 7 sites across England, Scotland and Northern Ireland. Heterogeneity of service provision was reported; however, this was unable to truly reflect the potential differences in care. | Yes – primary and secondary outcomes clearly articulated. Statistical analyses undertaken to explore impact of covariates on intervention effectiveness. | Yes – odds ratio, 95% confidence intervals and p value reported | Yes |
| **Tappin D. et al.** (2015) *Financial incentives for smoking cessation in pregnancy: Randomised controlled trial.* <https://doi.org/10.1136/bmj.h134> | Yes | Yes | Yes - flow diagram provided. Study benefitted from very low dropout rate following random assignment. However, more participants in the control group were lost to follow up at postnatal follow up | Yes – staff and participants were blind to allocation | Yes – however, control group participants had slightly higher nicotine dependence levels | Yes – recruitment of participants and care was given from a single site. | Yes –impact of different rates of lost to follow up between intervention and control group explored. ‘Gaming’ also explored. | Yes – odds ratio, 95% confidence intervals and p value reported | Yes – economic evaluation accompanied study which showed positive benefits of intervention over cost. |

**Table 4 – CASP checklist responses (systematic review)**

| **Study** | Did the review address a clearly focused question | Did the authors look for the right type of papers? | Do you think all the important, relevant studies were included? | Did the review’s authors do enough to assess quality of the included studies? | If the results of the review have been combined, was it reasonable to do so? | What are the overall results of the review? | How precise are the results? | Were all important outcomes considered? | Are the benefits worth the harms and costs? |
| --- | --- | --- | --- | --- | --- | --- | --- | --- | --- |
| **Campbell K. et al.** (2020) *Factors influencing the uptake and use of nicotine replacement therapy and e-cigarettes in pregnant women who smoke: a qualitative evidence synthesis.*  <https://doi.org/10.1002/14651858.CD013629> | Yes | Yes – qualitative and mixed methods studies included | Yes – comprehensive search strategy used | Yes – Wallace tool used. Process undertaken by two reviewers | Yes – qualitative synthesis was used to construct three overarching analytical themes developed from analysis. | Main finding was perceived harm to baby of NRT was one of the main reasons not to use the products. | N/A - qualitative synthesis only | Yes – conclusions drawn in relation to the relevance of the themes to both women’s decisions to start NRT and to their adherence to NRT in trials | Yes |

**Table 5 – checklist responses (mixed methods studies)**

| **Study** | Qualitative question or objective | Appropriate qualitative approach or design or method | Description of the context | Description of participants and justification of sampling | Description of qualitative data collection and analysis | Discussion of researcher's reflexivity | Appropriate sampling and sample (quantitative) | Justifications of measurements (validity and standards) | Controlling for confounding variables | Justification of the mixed methods design | Combination of qualitative and quantitative data collection-analysis techniques or procedures | Integration of qualitative and quantitative data or results | Score |
| --- | --- | --- | --- | --- | --- | --- | --- | --- | --- | --- | --- | --- | --- |
| **Cooper S. et al.** (2019) *Attitudes to E-cigarettes and cessation support for pregnant women from English stop smoking services: A mixed methods study.* <https://doi.org/10.3390/ijerph16010110> | 1 | 1 | 1 | 1 | 1 | 0 | 1 | 0 | 0 | 1 | 0 | 1 | 67% |
| **Thomson G. et al.** (2014) *Unintended consequences of incentive provision for behaviour change and maintenance around childbirth.* <https://doi.org/10.1371/journal.pone.0111322> | 1 | 1 | 1 | 1 | 1 | 0 | 1 | 1 | 0 | 1 | 0 | 1 | 75% |

**Table 6 – checklist responses (other study designs)**

| **Study** | Are the details of the methods used clearly reported, including reflection of the potential limitations of the method selected? | Is the study sample size, data collection and data analysis techniques appropriate for the objective of the study? | Are the conclusions drawn reasonable and justified in the context of the limitations of the method used? |
| --- | --- | --- | --- |
| **Campbell K.A. et al.** (2016) *'Opt-out' referrals after identifying pregnant smokers using exhaled air carbon monoxide: impact on engagement with smoking cessation support.* <http://dx.doi.org/10.1136/tobaccocontrol-2015-052662> | Yes – methods thoroughly reported and considerations to both the strengths and limitations of the study design were noted. Robustness of before-after study design was improved by using a fixed denominator, not prone to vary across time periods. | Yes – study sample size achieved was greater than the planned sample size. Statistical analysis to assess differences in key characteristics between time periods to improve precision of the reporting of intervention effectiveness. 95% confidence intervals reported. | Yes – findings are discussed in the context of the limitations of the design and in the context of existing literature and similar service evaluations. |
| **Bluegrass.** (2022) *Action on Smoking and Health: Qualitative insights.*  <https://ash.org.uk/uploads/Qualitative_Insights_Primary_Research_Report_2022-12-02-140553_trte.pdf?v=1669989950> | No – research was undertaken by focus group and in-depth interview. Details of how these were undertaken was not reported, nor how participants were recruited. Limitations of the methods were not discussed. | Yes – large number of participants involved with a broad mix of geographic areas, circumstances and range of ages. Study included smokers, quitters and lapsers to cover a broad experience. | Yes - themes from focus groups and interviews were identifiable. |
| **Forman J. et al.** (2017) *National survey of smoking and smoking cessation education within UK Midwifery School curricula.* <https://doi.org/10.1093/ntr/ntw230> | Somewhat – methods are well detailed, however reflections on limitations were only in respect of the questions asked and not the methods undertaken. | Somewhat – survey achieved a response of 55%. % of respondents to each question and response is reported. No statistical tests undertaken on data. | Yes – breadth of curricula is well reported, and findings are considered within the context of wider existing literature on experiences of smoking cessation services. |
| **Vaz L.R. et al.** (2017) Factors associated with the effectiveness and reach of NHS stop smoking services for pregnant women in England. <https://doi.org/10.1186/s12913-017-2502-y> | Yes – methods are clearly reported and limitations of using proxy data due to lack of available individual level data is reported. | Yes – study included 86% of usable survey responses. Range of statistical methods used to analyse data and sensitivity analyses undertaken to assess bias. P values reported. | Yes – findings are set out in the context of existing literature and potential limitations are well reported. |
| **Thomson R. et al.** (2022)  *Smoking Cessation Support for Pregnant Women Provided by English Stop Smoking Services and National Health Service Trusts: A Survey.* <https://doi.org/10.3390/ijerph19031634> | Somewhat – methods are well reported including design of separate surveys for different service delivery contexts. However, limitations of methods were not reported. | Somewhat – survey received a 70% response rate. Number and % of respondents to each question and response is reported. Reporting of responses across different settings allowed for some comparison (although not statistically) | Somewhat – comparison between sites and breadth of provision offers some insights into variable experiences of pregnant smokers. |
| **Local Government Association** (2018) *Fit for and during pregnancy: a key role for local government.*  <https://www.local.gov.uk/sites/default/files/documents/15.52%20Fit%20for%20and%20during%20pregnancy_03.pdf> | No – method and reflections on limitations not documented. | Somewhat – case studies are provided from a range of geographic areas. However, sample size within each area is very small. | Somewhat – successes are balanced with ‘lessons learnt’ from each site. |
| **Moyse M. et al.** (2020) Newspaper *media representation of electronic cigarette use during pregnancy.* <https://doi.org/10.1093/pubmed/fdaa048> | Somewhat – limited searching was undertaken. The potential impact of this is noted by the authors, but the rationale for not using a wider search strategy was not included. | Yes – analysis strategy was clearly reported and roles of the different authors in the process was clearly reported. | Somewhat – conclusions drawn were clearly anchored in the findings of the review, however the authors did not consider the findings in relation to existing wider literature about views of electronic cigarettes outside of media representation. |
| **Beenstock J. et al.** (2012)  *What helps and hinders midwives in engaging with pregnant women about stopping smoking? A cross-sectional survey of perceived implementation difficulties among midwives in the North East of England.* <https://doi.org/10.1186/1748-5908-7-36> | Yes – methods were clearly reported as well as potential limitations | Yes – although the survey response was small, and it was not clear how participants had been identified. The authors employed a range of statistical tests and drawn study conclusions. Results of statistical tests were reported with 95% confidence intervals and p values. | Yes – findings are set out in the context of existing literature. |
| **Bowker. K et al.** (2020)  *Pregnant women's use of e-cigarettes in the UK: a cross-sectional survey*. <https://doi.org/10.1111/1471-0528.16553> | Yes – methods were clearly reported as well as potential limitations, particularly the reliance on self-reported data. Baseline for study constructed from longitudinal study. | Yes – large sample size. Both screening and full questionnaires used to identify suitable participants. Results reported with 95% confidence intervals and p values for differences between groups. | Yes – findings are set out in the context of existing literature. |
